# Long COVID Syndrome and Takotsubo Cardiomyopathy: An Unwelcome Combination

**DOI:** 10.7759/cureus.17590

**Published:** 2021-08-31

**Authors:** Tahir Nazir, Hlaing Myat Chit Su, Paul Mann, Niall Clancy, Leila Kargar

**Affiliations:** 1 Internal Medicine, Lancashire Teaching Hospitals NHS Foundation Trust, Royal Preston Hospital, Preston, GBR; 2 Cardiology, Leighton Hospital, Crewe, GBR

**Keywords:** covid 19, cardiomyopathy, long covid syndrome, heart failure, broken-heart syndrome

## Abstract

Since the report of the first case from China in late 2019, the coronavirus disease (COVID-19) has spread very rapidly through the countries and regions leaving a trail of devastation in its path, everywhere. Although COVID-19 is primarily a respiratory illness mainly affecting the lungs; involvement of other organs including the cardiovascular system has been widely recognized. Whilst COVID-19 is an acute illness for a majority of cases; some of the debilitating virus-related symptoms can last for weeks and months, and are collectively termed as long COVID syndrome. Several published reports have described an association between acute COVID-19 illness and cardiac complications such as myocarditis and Takotsubo cardiomyopathy. However, little is known about any link between long COVID syndrome and the cardiac disease. We describe the case of a middle-aged woman with long COVID syndrome who presented with central chest pain and breathlessness. Her initial investigations showed an elevated cardiac troponin I and ischemic changes on 12 lead ECG. She was initially treated for non-ST elevation myocardial infarction. A subsequent coronary angiogram showed unobstructed coronary vessels and left ventricle (LV) gram demonstrated apical LV ballooning. She was managed conservatively and was discharged home following her clinical improvement. This case highlights the importance of holistic assessment of patients presenting with chest pain with the background of long COVID syndrome. It also outlines an emergent need to better understand pathophysiological mechanisms that underpin the development of cardiac complications in those with COVID-19 and long COVID syndrome.

## Introduction

Coronavirus disease 2019 (COVID-19), caused by severe acute respiratory syndrome coronavirus 2 (SARS-CoV-2), has led to significant mortality and morbidity worldwide, overwhelming the communities and healthcare systems alike. According to Johns Hopkins University of Medicine, Coronavirus Resource Centre, as of the last week of May, 2021, the total number of COVID-19 cases stands at 164 million with approximately 3.4 million fatalities globally [1]. Being a primary respiratory contagion, SARS-CoV-2 predominantly affects the lung parenchyma. However multisystem involvement due to either a direct viral invasion or immune-mediated tissue damage has been well described. Cardiac complications of acute COVID-19 such as myocarditis, pericarditis, pericardial effusion and Takotsubo cardiomyopathy have also been reported in the literature [2]. Long COVID syndrome is an increasingly known sequela of SARS-CoV-2 infection, which is characterized by a wide range of symptoms including shortness of breath, fatigue, chest pain, palpitations and lack of concentration to persist for several weeks and months after the index illness [3]. Whether long COVID syndrome also puts individuals at a higher risk of cardiac complications is not fully understood. We describe the case of a woman with long COVID syndrome who presented with Takotsubo cardiomyopathy mimicking an acute coronary syndrome.

## Case presentation

Our patient, a 58-year-old woman, presented to the emergency department with central chest pain. She described this as an acute severe retrosternal pain that radiated to the left shoulder and neck. It was associated with shortness of breath, nausea and diaphoresis. No emotional trigger could be identified in the history to account for her acute symptoms. Her past medical history included depression and COVID-19 six months previously. She had symptoms of ongoing tiredness, lethargy, lack of energy and breathlessness since recovering from COVID-19. There was no personal history of cardiac events, no family history of ischemic heart disease or sudden cardiac death. She was a lifelong non-smoker and was on regular citalopram 20 mg once daily.

On clinical examination, she was hemodynamically stable and had no peripheral oedema or calf swelling. On auscultation, her heart sounds were normal and there were bibasal fine crackles at both lung bases on auscultation.

**Figure 1:**
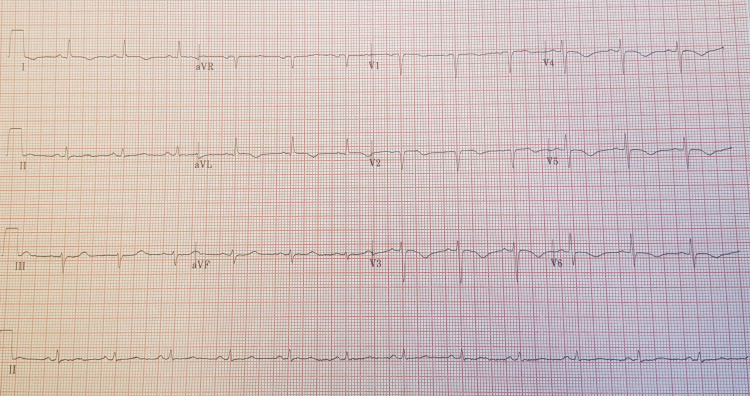
A 12 lead ECG shows sinus rhythm with a ventricular rate of 70 per minute, left axis deviation, T wave inversion V1 -V6.

**Figure 2:**
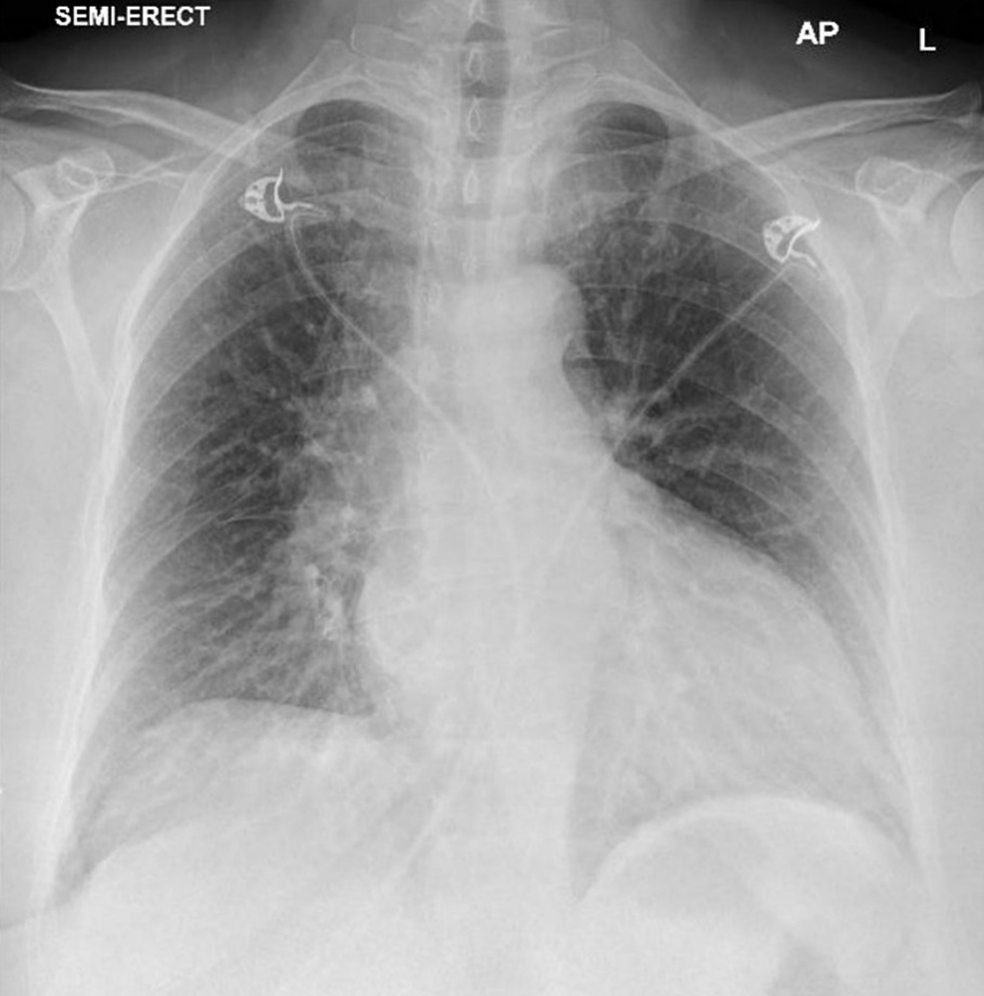
Semi-erect AP chest x-ray shows prominent pulmonary vasculature and congested lung fields.

Her 12 lead ECG showed sinus rhythm with a ventricular rate of 70 beats per minute, left axis deviation and T wave inversion in lead V1 to V6 (Figure 1). Her laboratory investigations revealed normal full blood count and renal function test with estimated glomerular filtration rate of >90 ml/min. Liver and bone profile and inflammatory markers were normal. Her cardiac troponin I was elevated 30 ng/L (normal range 5-14 ng/L) that rose to 3050 ng/L upon repeating after six hours. Her admission chest x-ray demonstrated prominent pulmonary vasculature and congested lung fields (Figure 2).

**Figure 3:**
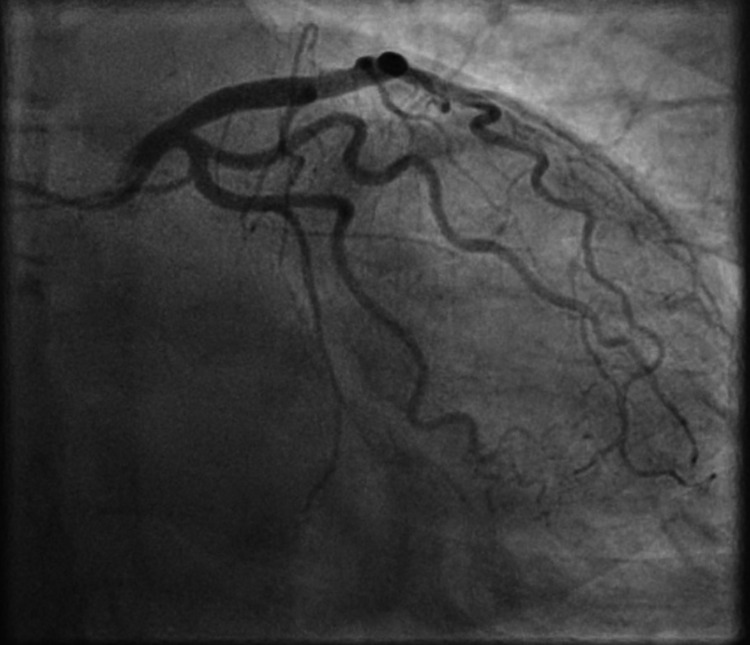
Invasive coronary angiography shows unobstructed left sided coronary arteries.

**Figure 4:**
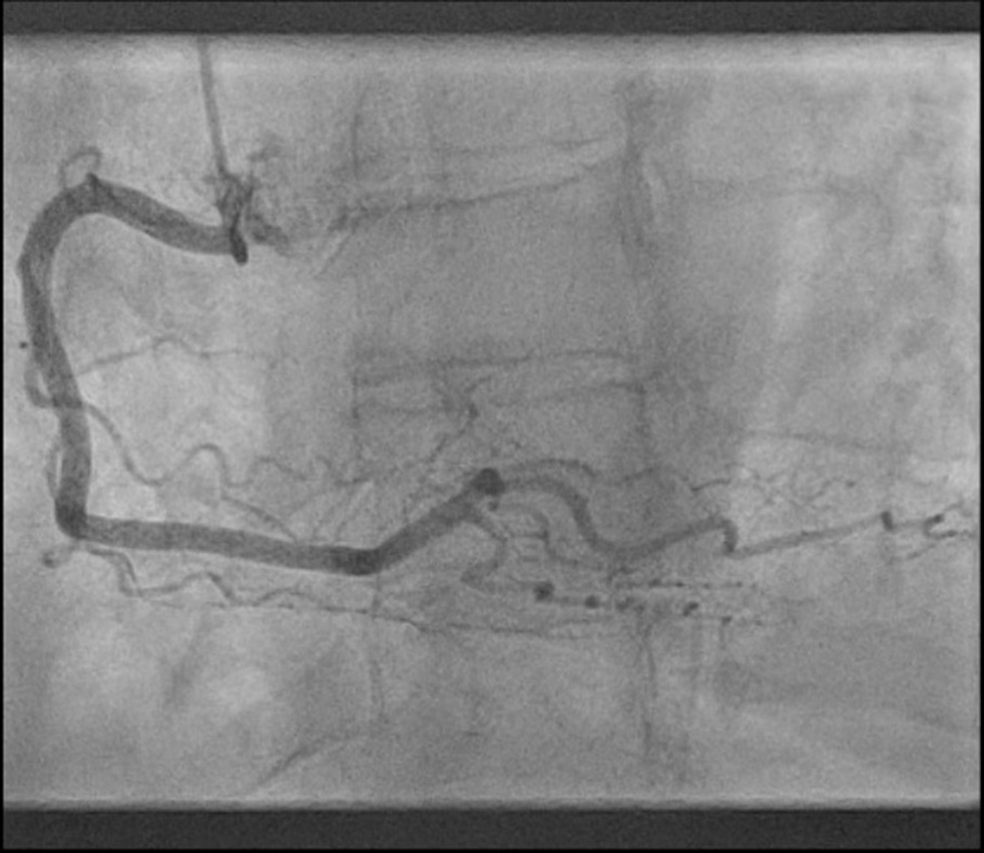
Invasive coronary angiography shows unobstructed right coronary artery.

She was treated with oral dual antiplatelets (aspirin and clopidogrel), subcutaneous fondaparinux, sublingual nitrates, intravenous opioid analgesia and loop diuretic (furosemide) and inhaled oxygen therapy. She was put on a cardiac monitor and was transferred to cardiology. Whilst in the cardiology unit, due to her ongoing symptoms of chest pain, she was commenced on the infusion of glyceryl tri-nitrate (GTN). However, her symptoms continued and she had to be urgently transferred to a regional cardiology centre for further management. Her coronary angiogram showed un-obstructed coronary arteries (Figures 3, 4).

**Figure 5:**
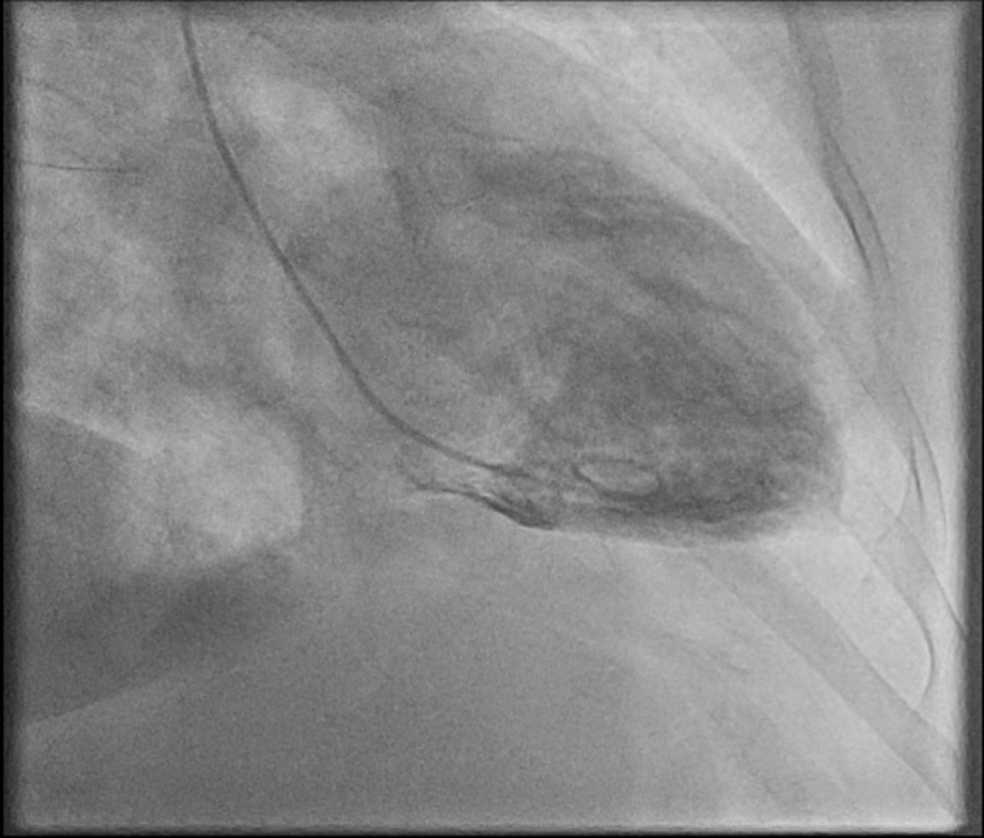
Invasive left ventricle (LV) gram shows ballooning of the left ventricle’s apical region.

Invasive left ventricle (LV) gram revealed systolic dysfunction and ballooning of the left ventricle apex, with an estimated LV ejection fraction of 45-50% (Figure 5).

**Figure 6:**
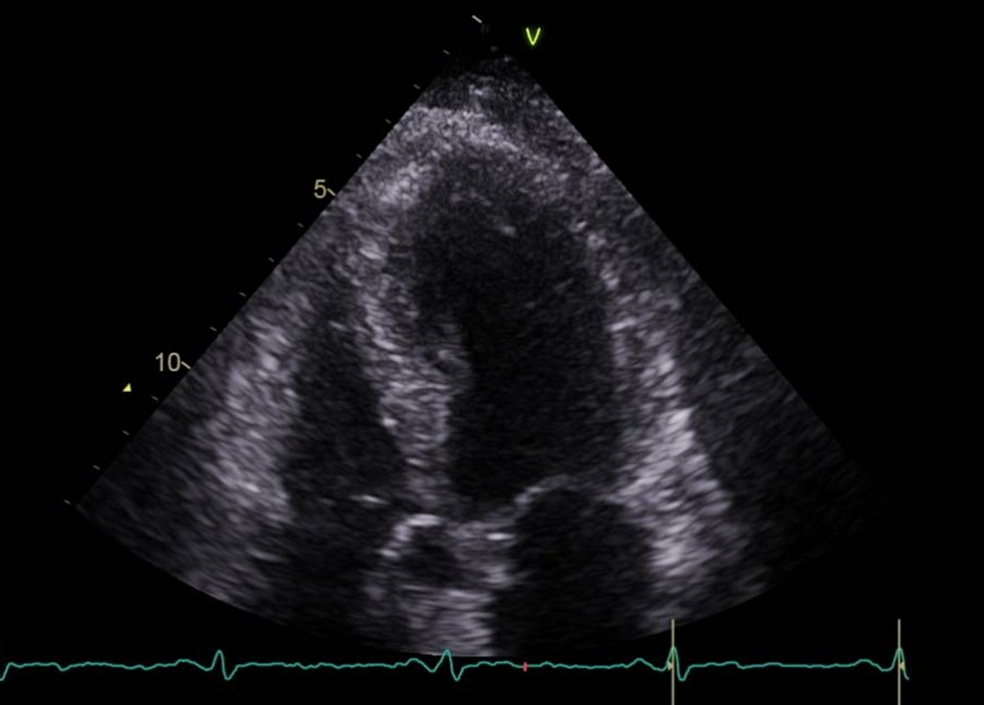
Transthoracic echocardiogram (modified 4 chamber view with a left ventricle close up) reveals some ballooning of the left ventricle (LV) apex.

A subsequent transthoracic echocardiogram confirmed the mild impairment of left ventricular systolic function along with apical ballooning with an LV ejection fraction of 50% (Figure 6).

**Figure 7:**
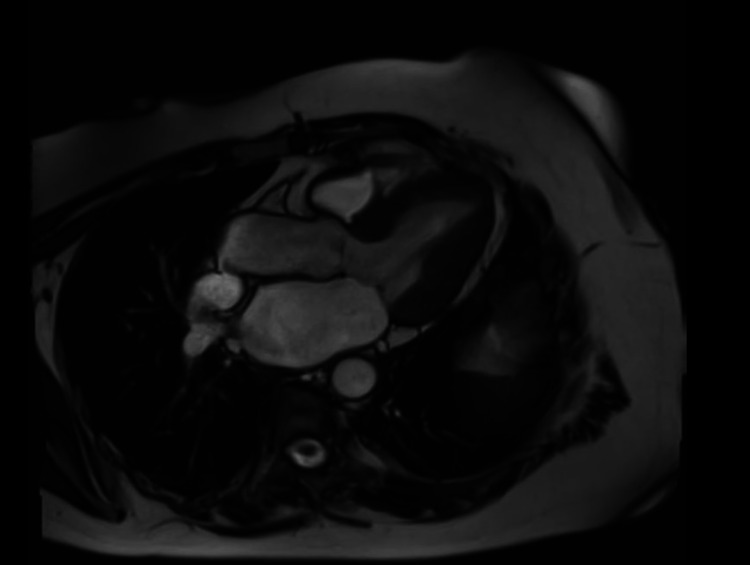
Cardiac MRI scan performed 3 months after discharge from hospital shows normal contractility of the left ventricle, along with resolution of apical ballooning.

Following this, her condition started to improve, and symptoms of chest pain and shortness of breath settled. A repeat 12 lead ECG prior to her discharge home showed sinus rhythm, ventricular rate of 64 per minute, left axis deviation and persistence of T wave inversion in precordial leads V1-V6 (similar to her admission ECG). Due to the lack of any LV outflow tract obstruction on the echocardiogram and having no intra-cavity gradient in the LV, we stopped her beta-blocker treatment. She was discharged home following the resolution of her symptoms on aspirin and losartan (angiotensin receptor blocker). A cardiac MRI scan was performed three months after her discharge which demonstrated that left ventricular function had returned to normal and apical ballooning had resolved (Figure 7).

## Discussion

The destructive impact COVID-19 has left in its wake globally is clear for all to see, none more so than in the health sector which has been dealt the harshest of blows [4]. Whilst COVID-19 is primarily a respiratory condition, its impact on the organ systems should not be underestimated. Published reports have widely described COVID-19’s cardiac sequelae, which include inflammation of the myocardium and pericardium, arrhythmias, myocardial infarction and left ventricular systolic dysfunction [5]. Emerging data reveals the incidence of cardiac complications in COVID-19 patients to range from 19-28% and is associated with poorer clinical outcomes. Takotsubo cardiomyopathy, also termed 'broken heart syndrome' is an increasingly recognised reason for acute left ventricular dysfunction [6,7]. Recent literature has also highlighted potential links between acute COVID-19 and Takotsubo cardiomyopathy; a systematic review of 12 such cases has shown its incidence to be the highest in females in the fifth and sixth decade of life [8]. This is similar to the incidence of Takotsubo cardiomyopathy in the general population without concurrent COVID-19.

A catecholamine surge, often precipitated by overwhelming physical or emotional stress, is considered to be the main trigger leading to Takotsubo cardiomyopathy. A plausible mechanistic explanation involves a sudden increase in serum cortisol and catecholamine levels resulting in myocardial stunning, microvascular dysfunction, poor myocardial relaxation, increased isometric tension and eventually myocardial systolic dysfunction [9]. Incidentally, accumulating evidence suggests that in patients with COVID-19, severe systemic inflammation and hypoxaemia also lead to an acute surge of catecholamines and cytokine storm creating a hostile microenvironment for cardiomyocytes predisposing the heart to the development of Takotsubo cardiomyopathy [10].

Clinical features of acute COVID-19 have been well publicised and comprise fever, cough, shortness of breath, anosmia and myalgia. As the disease progresses and pulmonary infiltrates develop, hypoxia, respiratory failure, haemodynamic collapse and multiorgan damage ensue [11]. Those with concurrent Takotsubo cardiomyopathy also have breathlessness, orthopnoea, chest pain, palpitations and fever [12]. In many cases, the presenting features may be identical to a myocardial infarction with clinical heart failure. A stressful event other than COVID-19 infection may also be evident in the clinical history. Patients with long COVID syndrome experience a broad spectrum of troublesome symptoms for weeks and months after the initial infection. High levels of anxiety and stress have been reported in the sufferers of long COVID syndrome which probably offer a substrate for the development of Takotsubo cardiomyopathy.

Laboratory investigations may show an elevated white blood cell count with relative lymphopenia, raised inflammatory markers such as C-reactive protein and interleukin-6. In addition, cardiac troponin and pro-brain natriuretic peptide (BNP) levels are also frequently raised. Electrocardiogram usually shows wide-spread T wave inversion, although ST segment elevation and QT interval prolongation have also been reported. Chest x-ray may show pulmonary infiltrates due to COVID-19 with or without pulmonary congestion and pleural effusions due to heart failure. An echocardiogram reveals a variable degree of systolic dysfunction with regional wall motion abnormalities not confined to a single vascular territory. Classical apical and mid-segment akinesia with ballooning is the most common form of Takotsubo cardiomyopathy in the general population as well as in those with COVID-19 illness [13]. As a result, ventricular basal segments may become hyperkinetic and LV outflow tract obstruction may be seen in nearly a quarter of patients. Left ventricular ejection fraction is usually low. Inverted Takotsubo cardiomyopathy is a rare variant that is characterised by basal hypokinesia and apical hypercontractility [14]. Cardiac catheterization is routinely carried out and usually reveals un-obstructed coronary vessels with typical appearances of LV gram. Cardiac MRI scan is useful in ruling out differential diagnoses such as myocarditis [15].

Initial management is often carried antiplatelet medications and beta-blockers whilst further investigations are being performed to rule out myocardial infarction. Diuretics and appropriate heart failure medications are usually required in the acute phase of the illness. Supportive treatment with beta-blockers may be useful in those with an element of LV outflow tract obstruction. Reassuringly, in a majority of patients, Takotsubo cardiomyopathy has an excellent prognosis, and the condition resolves spontaneously [16]. Clinical signs and symptoms settle, ECG changes normalise, and the left ventricular systolic function is restored back to normal.

## Conclusions

It is interesting to note that cardiovascular comorbidities account for the poor prognostic risk factors for COVID-19, however COVID-19 itself can cause cardiovascular injuries in many mechanisms. Stress-induced cardiomyopathy is one of them and on a rising trend during the COVID-19 pandemic. Clinicians should be more aware of this differential diagnosis in patients presenting with chest pain and shortness of breath during the acute phase or the recovery period of COVID-19.
